# Is It Just About Scrolling? The Correlation of Passive Social Media Use with College Students’ Subjective Well-Being Based on Social Comparison Experiences and Orientation Assessed Using a Two-Stage Hybrid Structural Equation Modeling–Artificial Neural Network Method

**DOI:** 10.3390/bs14121162

**Published:** 2024-12-04

**Authors:** Ziyu Liu, Liyao Xiao

**Affiliations:** Department of Media Content, Cheongju University, Cheongju-si 28503, Republic of Korea

**Keywords:** social media use, social comparison experiences, social comparison orientation, subjective well-being, two-stage hybrid SEM-ANN

## Abstract

Previous studies have found that passive social media use (PaSMU) tends to induce upward contrast, thereby affecting well-being. However, this perspective alone may overlook the mechanisms of other social comparison phenomena. This study analyzes the influence mechanism of PaSMU on subjective well-being (SWB) by categorizing social comparison into upward identification, upward contrast, downward identification, and downward contrast while incorporating social comparison orientation (SCO) as a moderating variable. This study surveyed college students who use RED (Xiaohongshu) and collected 352 valid questionnaires. A two-stage hybrid structural equation modeling (SEM)–artificial neural network (ANN) method was employed, utilizing path and mediation effect analysis to verify the moderating effect of SCO in the process of PaSMU affecting SWB. PaSMU is positively correlated with upward contrast and downward identification, both of which negatively affect SWB. Upward contrast and downward identification are associated with lower SWB, while downward comparison is positively correlated with SWB. High SCO strengthens the association between upward contrast and reduced SWB. Furthermore, upward contrast and downward identification were found to have comparable mediating effects between PaSMU and SWB. In contrast to previous studies, this research highlights that downward identification plays a comparably significant mediating role alongside upward contrast. Downward identification significantly mediates the relationship between PaSMU and SWB due to increased risk awareness, higher sensitivity to negative information among socially anxious students, emotional contagion from negative content, and anonymity that fosters an “imagined community”. Additionally, students with high SCO are more affected by idealized self-presentations and rely on upward contrasts for social feedback, lowering their SWB. This study reveals the complex correlation of PaSMU and SWB, providing new theoretical insights and practical strategies to encourage positive social media use among college students.

## 1. Introduction

In the past decade, the use of social media has shown exponential growth [1], with social media playing an increasingly significant role in information access, maintaining social connections, self-expression, and emotional support [2]. Primary users are constantly influenced by social media in terms of their psychology and behavior [3]. Since Facebook and Instagram are not accessible in China, a local alternative called RED (Xiaohongshu) has emerged. Since its launch in 2014, RED has become widely popular among college students, who use the platform to search for information, post text, photos, and short videos, and interact with others through likes, comments, and shares. As of July 2019, RED’s registered users exceeded 300 million, with daily exposure reaching 3 billion views [4]. Although the increasing features of similar social media platforms have brought positive effects on life, the rapid growth in user numbers and usage time has raised public concerns about their potential impact on college students’ psychology and behavior.

A substantial body of empirical research has investigated the impact of social media usage on subjective well-being (SWB) of college students; however, these studies have not yet reached consistent conclusions [2]. On the one hand, accurately assessing this concept is challenging due to significant differences in how social media usage is defined. For example, measuring social media usage by time or frequency has limitations, as people often find it difficult to accurately report frequency or duration [5]. Additionally, time-based measurements may introduce the issue of the displacement hypothesis, limiting engagement in other activities beneficial to well-being [6]. This has led more researchers to recognize that the impact of social media on SWB largely depends on users’ engagement style and the specific content they encounter rather than merely on duration or frequency [7]. To address this issue, Burke et al. [8] proposed the active–passive social media use (ASMU-PaSMU) dichotomy. Passive social media use (PaSMU) refers to instances where users acquire information without engaging in interactions, such as browsing and viewing others’ content [9]. Verduyn et al. [10] found that PaSMU is negatively correlated with SWB, with a more pronounced and stable impact compared to active use. Meier and Krause [11] noted that due to the complexity of how social media influences users, introducing mediating and moderating variables is essential for deeper analysis.

Many studies argue that social media use influences social comparison, which subsequently has a negative impact on SWB [12,13]. They suggest that individuals tend to compare themselves to those who appear to perform better, leading to lowered self-evaluation, feelings of envy, and a decline in overall mood. However, focusing solely on upward contrast has certain limitations. Given the diverse forms of interaction on social media, users may not only experience upward contrast but may also engage in other types of social comparison. Additionally, early studies simply associated upward contrast with negative emotions and downward comparison with positive emotions, overlooking situations where both types could simultaneously evoke both positive and negative emotions [14]. This is because perceived control can also serve as an additional dimension in differentiating social comparisons [15]; that is, even within the same direction for comparison, the level of control individuals feel in narrowing the gap with their comparison targets can influence the outcome of the comparison. For example, when college students see others achieving outstanding academic results on RED, if they believe they can attain similar achievements through effort, their self-identity is likely to strengthen; conversely, if they feel unable to achieve those results despite their best efforts, it may increase their risk of depression [16]. Therefore, when applying a social comparison framework, beyond categorizing comparison targets into upward (others who are superior) and downward (others who are inferior), social comparison outcomes should be further refined into two dimensions: identification and contrast. In other words, upward social comparison experiences can be divided into upward identification and upward contrast, while downward social comparison experiences can be divided into downward identification and downward contrast, thus resulting in four distinct types.

In addition to the direction of social comparison, some studies have found that personality differences also influence the emotional effects arising from social comparison experiences [17], with social comparison orientation (SCO) being a prominent factor. SCO refers to an individual’s tendency, when faced with uncertainty, to be sensitive to and reliant on others’ behaviors rather than personal beliefs or convictions and to respond accordingly [18]. The effects of social comparison vary based on the SCO of individuals. Park and Baek [18] found that users with higher SCO are more likely to experience decreased well-being from upward contrast and increased well-being from downward identification.

This study differs from previous ones by focusing on PaSMU among college students and subdividing social comparison experiences into four types: upward identification, upward contrast, downward identification, and downward contrast. It introduces SCO as a moderating variable and examines the distinct emotional and cognitive impacts of these variables on SWB. Additionally, based on hybrid SEM-ANN analysis, the reliability of the findings is enhanced. This study not only reveals the multidimensional mechanisms of social comparison experiences in social media use but also provides a new theoretical framework for understanding the SWB of college students. Furthermore, it offers practical guidance for educators and developers of social media platforms and supports the design of intervention strategies that foster mental health and encourage a positive usage of social media, thereby contributing to a more favorable social interaction environment.

## 2. Literature Review and Hypotheses

### 2.1. PaSMU and SWB

Although extensive research has accumulated on media use and SWB, most studies primarily focus on the effects of duration and frequency of social media usage on SWB. Some scholars have found that social media can provide online support, boost self-esteem, and increase social capital, thereby positively affecting SWB [19]. However, other studies suggest that excessive social media use may lead to internet addiction, reduce SWB, and trigger psychological issues such as anxiety and depression [20,21]. In addition, an eight-year study of 500 adolescents showed no significant impact of social media use on mental health [22]. Due to these inconsistent findings, researchers have increasingly focused on active and passive social media use as key variables for examining the patterns of social media use [23].

PaSMU refers to the process of obtaining information without engaging in interactions, such as browsing and viewing others’ content [9]. Verduyn et al. [10] show that PaSMU is negatively correlated with SWB and exerts a more significant and stable influence than active use. Many scholars support this conclusion; for example, Thorisdottir et al. [9] indicated that PaSMU is more associated with severe anxiety and depressive symptoms among adolescents. By integrating active and passive use within a single model, Han et al. [24] emphasized that passive use has a more common and pronounced association with depression. However, some studies have suggested that passive social media use (PaSMU) may enhance well-being and reduce the incidence of mental health issues [25]. A recent review of 36 studies further revealed the heterogeneity in findings regarding the relationship between PaSMU and well-being. The review highlighted that previous definitions of PaSMU were overly simplistic, making it challenging to accurately capture its relationship with well-being or mental health [26]. Given the complexity of the mechanisms underlying passive social media use, incorporating mediating and moderating variables is essential for more nuanced and in-depth analysis [11].

Some studies categorize PaSMU into public and private types [27]. Given that users often employ social media for private communication [28], private PaSMU is defined as interactions with known contacts within one-on-one or small group settings. Earlier empirical studies primarily focused on public PaSMU, suggesting that exposure to content from less close connections (such as celebrities or influencers) may reduce well-being [27]. In contrast, private PaSMU, which usually involves close friends or family, may positively affect well-being [29]. As one of China’s major social media platforms, RED allows users to acquire information by browsing content related to life experiences, daily routines, and practical tips, with relatively low levels of active engagement. This passive browsing is highly prevalent among college students. Additionally, RED permits users to follow any registered account, whether public or private. Thus, public and private PaSMU are deeply intertwined. Consequently, this study takes into account the high integration between public and private PaSMU, refraining from distinguishing them separately to more comprehensively reflect actual user behavior on the RED platform and its potential impact on individuals. Based on the analysis above, this study proposes the following hypothesis:

**H1.**  *PaSMU has a negative impact on the SWB of college students.*

### 2.2. Four Types of Social Comparison Experiences

Social comparison refers to the process by which individuals evaluate their performance in relation to others or decide how they should behave, think, and feel. This comparison not only helps us understand our abilities and social status relative to others, thereby facilitating better adaptation to society, but it also fulfills basic human needs such as belongingness and self-esteem. Consequently, it is recognized as a significant mediating variable between social media use and SWB [14]. Social comparison can be categorized into upward and downward types, with users experiencing different social comparison outcomes based on reference groups [30]. Research indicates that in the context of social media, users are prone to engage in upward contrast, which is related to the inherent characteristics of social media. For instance, Fan et al. [31] suggest that the primary purpose of most posts on social media is to present oneself in an enhanced manner. In particular, individuals with strong narcissistic tendencies often showcase their superior lifestyles through self-promotional posts.

Existing research indicates that social comparison on social media has a significant correlation on users’ psychological well-being. Some studies suggest that social media use may negatively influence SWB by amplifying social comparison [12,13]. Users often tend to compare themselves with individuals who appear more successful, leading to lower self-evaluations, feelings of envy, and overall emotional deterioration [32]. However, other studies argue that social comparisons on social media do not always yield negative outcomes. For instance, comparisons focused on opinions rather than abilities typically do not result in negative emotions [33], and downward comparisons tend to have minimal impact on SWB [34]. Recent research further reveals that viewing social media influencers with demographic similarities may increase upward comparisons, yet this has negligible effects on well-being [35]. However, when users observe others living vibrant lives, some may feel a sense of relative deprivation, while others may experience positive emotions inspired by aspirations and expectations of success [36]. These findings suggest that focusing solely on upward comparisons may be limiting, and the diverse interaction forms and multidirectional comparisons on social media warrant deeper exploration.

Moreover, responses to comparison targets are equally important. Most prior studies have heavily emphasized the potential negative consequences of upward social comparison and narrowly focused on upward comparison emotions, such as envy or depression [18]. Adopting a contrast strategy during upward comparison often triggers negative emotions such as shame or envy, whereas adopting assimilation can evoke a sense of inspiration, positively influencing SWB [18]. In contrast, downward comparison tends to elicit positive emotions such as pride or schadenfreude, while downward assimilation may evoke negative emotions such as worry or sympathy [37,38]. This classification approach provides a clearer understanding of the relationship between social media use and well-being, as users’ reactions can lead to varying psychological outcomes.

**Figure 1:**
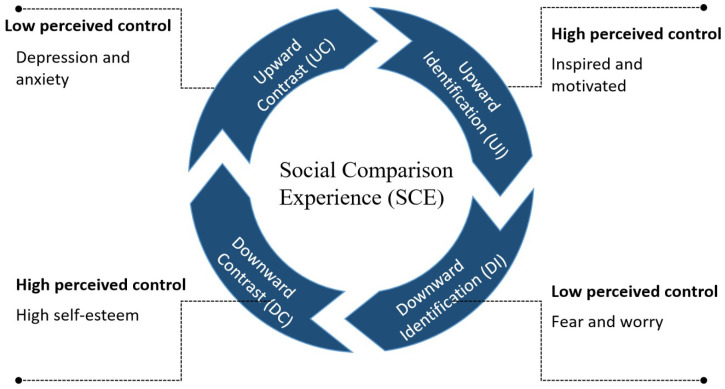
Four types of social comparison experiences.

We further conceptualize this social comparison experience into two dimensions: identification and contrast. In contrast to existing research, this study redefines the social comparison experiences in PaSMU into four types, i.e., upward contrast, upward identification, downward contrast, and downward identification (Figure 1), focusing on the impact of these four distinct types of social comparison experiences on SWB of college students. Based on the above analysis, this study proposes the following hypotheses:

**H2.**  *PaSMU has a positive impact on upward contrast.*

**H3.**  *PaSMU has a negative impact on upward identification.*

**H4.**  *PaSMU has a negative impact on downward contrast.*

**H5.**  *PaSMU has a positive impact on downward identification.*

Previous research has indicated that different types of experiences yield varying emotional outcomes; for instance, upward identification typically generates positive emotions such as inspiration and optimism, whereas upward contrast may lead to negative emotions like jealousy and depression. Likewise, while downward contrast can evoke positive feelings like pride, it may also be associated with negative emotions such as fear and anxiety in certain contexts [39]. Consequently, this study proposes the following hypotheses:

**H6.**  *Upward contrast has a negative impact on the SWB of college students.*

**H7.**  *Upward identification has a positive impact on the SWB of college students.*

**H8.**  *Downward contrast has a positive impact on the SWB of college students.*

**H9.**  *Downward identification has a negative impact on the SWB of college students.*

Given that social identification has been extensively studied and confirmed as a mediating factor between social media use and SWB, this study proposes the following hypotheses:

**H10.**  *Upward contrast mediates the relationship between PaSMU and the SWB of college students.*

**H11.**  *Upward identification mediates the relationship between PaSMU and the SWB of college students.*

**H12.**  *Downward contrast mediates the relationship between PaSMU and the SWB of college students.*

**H13.**  *Downward identification mediates the relationship between PaSMU and the SWB of college students.*

### 2.3. SCO

SCO refers to the tendency to compare one’s achievements, circumstances, and experiences with those of others. Currently, there is limited research on the impact of SCO on mental health [40]. Previous studies have distinguished between two types of SCO, as they have different effects on mental health. Users with a strong ability-based SCO tend to view others as competitors and compare themselves to their friends. If they perceive themselves as superior to their friends, their well-being may improve; conversely, if they feel inferior, their well-being may decline [41,42]. In contrast, users with a strong opinion-based SCO tend to regard others as sources of reference, learning from their successes or failures; thus, their SWB is less likely to be affected by upward contrast [18]. Regardless of whether the comparison is ability-based or opinion-based, research consistently shows that SCO plays an important moderating role between social comparison experiences and SWB of college students. Park and Baek [18] argue that if a user’s SCO activates social comparison experiences of upward identification or downward contrast, their SWB may improve. However, if it activates upward contrast or downward identification, negative effects may occur. In other words, the SCO of college students can predict whether the relationship between the four social comparison experiences and SWB is positive or negative. Wang et al. [43] validated the moderating effect of SCO between PaSMU and upward contrast using structural equation modeling (SEM). To further refine this conclusion, we examine whether SCO moderates the relationship between upward contrast and SWB. Therefore, this study proposes the following hypothesis:

**H14.**  *SCO moderates the relationship between upward contrast and the SWB of college students.*

### 2.4. Research Framework

**Figure 2:**
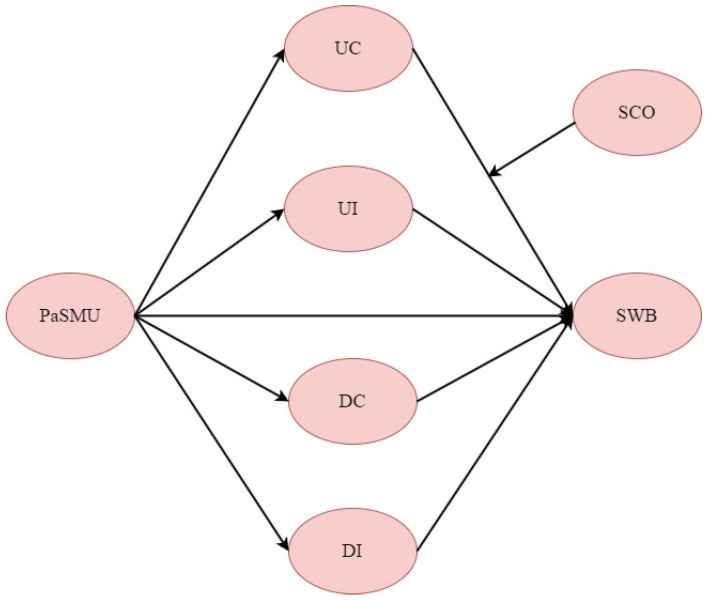
Research framework.

This study surveyed college students to construct a hypothetical model that includes PaSMU as the independent variable, four mediating variables, i.e., upward contrast (UC), upward identification (UI), downward comparison (DC), and downward identification (DI), SWB as the dependent variable, and SCO as the moderating variable (Figure 2). The results of this study contribute to a deeper understanding of the impact of social media use on the mental health of college students, providing a more complex analytical framework that demonstrates how specific patterns of social media use relate to SWB and how these impacts vary based on individual tendencies of social comparison. This study aims to lay a foundation for further exploration of the relationship between social media use and the well-being of college students from a more complex perspective.

## 3. Research Methods

### 3.1. Research Process and Steps

This study uses an online questionnaire survey. After establishing the theoretical model, the hybrid SEM-ANN approach is used to evaluate the model and hypotheses. The specific steps include (1) proposing the model and hypotheses through an analysis of prior studies, (2) designing a questionnaire based on all hypotheses and surveying RED users among college students, (3) conducting SEM analysis on the questionnaire data to validate the model and hypotheses, (4) introducing the variables and their data into ANN analysis based on the hypotheses supported by SEM results to measure the stability of the results and the normalized importance of independent variables, and (5) discussing the results and their implications.

### 3.2. Data Collection and Questionnaire Development

This study utilized the Wenjuanxing platform (www.wjx.cn) to design an online questionnaire and commissioned its distribution to college students who are users of RED. Wenjuanxing is a professional online academic survey platform specializing in services such as questionnaire design and data collection. To encourage participation, small electronic gift cards and opportunities to enter a prize draw were offered as incentives. Additionally, the questionnaire included an introductory section that clearly explained the purpose of this study, assurances of anonymity, and data confidentiality commitments to build trust and motivate participants to provide thoughtful responses. To ensure data quality, screening mechanisms were implemented, such as filtering out invalid responses based on response time. Ultimately, high-quality samples meeting this study’s criteria were retained for analysis. Between June and July 2024, a total of 428 questionnaires were distributed to Chinese college students who had used RED. Incomplete or invalid responses (those outside the survey scope and with response times under 90 s) were excluded, resulting in 352 valid questionnaires for analysis. Among the respondents, 157 were male (44.6%) and 195 were female (55.4%). The age distribution was as follows: 27 respondents aged 18 (7.68%); 81 respondents aged 19 (23.01%); 79 respondents aged 20 (22.44%); 83 respondents aged 21 (23.58%); 49 respondents aged 22 (13.92%); and 33 respondents aged 22 and above (9.38%). For education background, 319 were undergraduate students (90.63%), 20 were master’s students (5.68%), and 13 were doctoral students (3.69%). The distribution of years spent in school was as follows: 79 respondents with less than 1 year (22.44%); 81 respondents with 1–2 years (23.01%); 78 respondents with 2–3 years (22.16%); 70 respondents with 3–4 years (19.89%); 31 respondents with 4–5 years (8.81%); and 13 respondents with more than 5 years (3.69%).

The survey included a total of 7 latent variables with 27 items, all utilizing a 7-point Likert scale. The questionnaire was translated into Chinese in advance to ensure the accuracy of the content. To avoid the limitations of operationalizing social media use only through duration or frequency, we referenced and modified the scale designed by Ding et al. [44] and selected 5 items to examine PaSMU. The four types of social comparison experiences were based on the measurement tools in Batenburg and Das [39] and were modified and supplemented, covering upward contrast, upward identification, downward comparison, and downward identification, with 3 items dedicated to each type, totaling 12 items. To measure SWB, we used the life satisfaction scale developed by Diener et al. [45] and the affect scale developed by Watson et al. [46], selecting and modifying 6 items from these tools. These scales are standardized tools for measuring SWB and have received multiple validations. The SCO variable was based on the scale developed by Park and Baek [18] and was modified to include 4 items. All scales employed in this study have demonstrated good reliability and validity in previous research, providing a solid foundation for the construction of the model for this study.

### 3.3. Two-Stage Hybrid SEM-ANN Approach

In investigating the impact of PaSMU on the SWB of college students, traditional linear methods such as multiple regression analysis (MRA) and SEM are effective for linear relationships. However, given that this study includes four mediating variables and one moderating variable, these methods have limitations. To address the constraints of linear assumptions, this research is based on SEM and introduced multilayer perceptron (MLP), which is a specific type of artificial neural network (ANN), creating a more precise predictive framework. Unlike traditional linear approaches, ANN can identify nonlinear and non-compensatory relationships [47]. With a two-stage hybrid SEM-ANN approach, this study captures the complex relationship between PaSMU and SWB more effectively, enhancing predictive capability and providing rigorous empirical support for understanding the factors influencing the mental health of college students.

## 4. Research Results

### 4.1. Results of Reliability, Validity, and Structural Equation Modeling

Data analysis for this study was conducted using SPSS 26 and AMOS 26. Reliability was assessed through Cronbach’s α and factor loadings, while AVE indicates the extent to which latent variables explain the variance of the indicators, and CR represents the consistency of the variables. A factor loading greater than 0.5 signifies adequate reliability. In this study, all factor loadings exceeded 0.5, and all AVE values were above 0.5, indicating good convergent validity. Additionally, both Cronbach’s α and CR values surpassed 0.7, confirming the internal consistency of the latent variables (Table 1).

To further assess validity, this study conducted a correlation analysis among the factors. The results indicated that the square roots of AVE for each latent variable were greater than the correlation coefficients among the latent variables, demonstrating that this model has strong discriminant validity (Table 2).

This study utilized AMOS 26 to construct the SEM and validated the model fit using key indicators such as CMIN/DF, GFI, AGFI, CFI, and RMSEA. The results revealed that the value of CMIN/DF was 2.174 (satisfying the criterion of 1 < CMIN/DF < 3), with GFI, CFI, and AGFI values of 0.843, 0.908, and 0.814, respectively. Although GFI was slightly below the desired level, it remained within an acceptable range. The value of RMSEA was 0.058, which satisfied the standards and confirmed the model’s applicability (Table 3).

### 4.2. Analysis of Mediating Effects

Since the model includes multiple mediating variables, the mediating effects are relatively complex. To better understand this process, we measured and compared the specific indirect effects across different pathways. If the indirect effect between the independent variable and the dependent variable is significant while the direct effect is not, it indicates full mediation; if both the direct and indirect effects are significant, it indicates partial mediation. This study employed the bootstrapping method in AMOS to demonstrate the existence of partial mediation in the model, as the direct effect between the independent variable PaSMU and the dependent variable SWB was significant (*p* < 0.05), and the indirect effects were also significant (*p* < 0.05) after introducing the mediating variables (upward contrast, upward identification, downward comparison, downward identification). The specific mediating variables and their effect strengths require further analysis of the specific indirect effects (Table 4).

This report shows that among the mediating variables between PaSMU and SWB, the pathways of upward identification and downward comparison did not reach significance (*p* > 0.05), while the other specific indirect effects were significant (*p* < 0.05). After excluding upward identification and downward comparison, the strengths of the specific indirect effects of the remaining mediating variables were as follows: upward contrast (41.2%) > downward identification (39.1%). There was a significant difference between the pathways of upward contrast and upward identification (*p* < 0.05), while no significant differences were found among the other specific indirect effects (*p* > 0.05).

### 4.3. Analysis of Moderating Effects

This study further examined the moderating role of SCO in the relationship between upward contrast and life satisfaction. Specifically, SCO was divided into high and low groups based on the mean value to observe changes in the impact of upward contrast on SWB. The path analysis indicated that the interaction term between upward contrast and SCO was significantly negatively correlated with SWB (*β* = −0.145, *p* < 0.01, see Table 5). Bootstrap in AMOS was used to calculate simple effects, the results revealed that among college students in the high SCO group (mean + 1 SD), the association between upward contrast and SWB was significant (*β* = −0.38; BC 95% CI = [−0.599, −0.170]; *p* < 0.001); whereas in the low SCO group (mean − 1 SD), the association was not significant (*β* = 0.040; BC 95% CI = [−0.155, 0.242]; *p* > 0.05, see Table 6).

**Figure 3:**
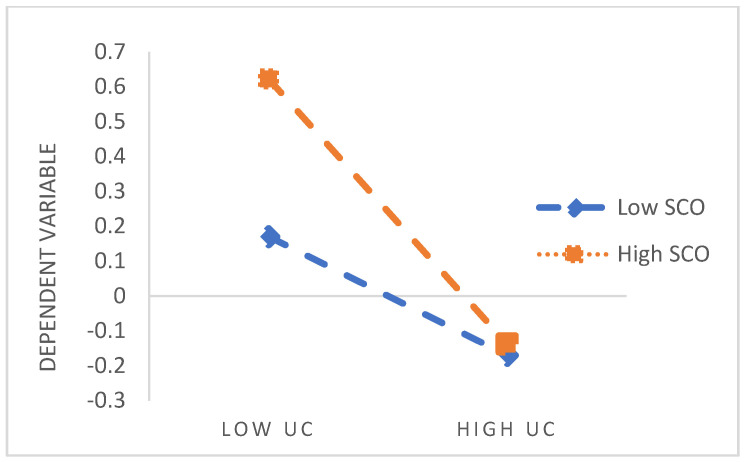
Interaction of SCO with UC and SWB.

Following the recommendations of Cohen et al. [48] regarding the interpretation of the moderating effects of two predictor variables on the dependent variable, we plotted the interaction between upward contrast and SWB (Figure 3). The results indicate that individuals in the high-SCO group experience a significant decrease in SWB when engaging in upward contrast compared to those in the low-SCO group. Therefore, SCO clearly serves as an amplifying moderator between upward contrast and SWB.

### 4.4. Hypothesis Test

**Figure 4:**
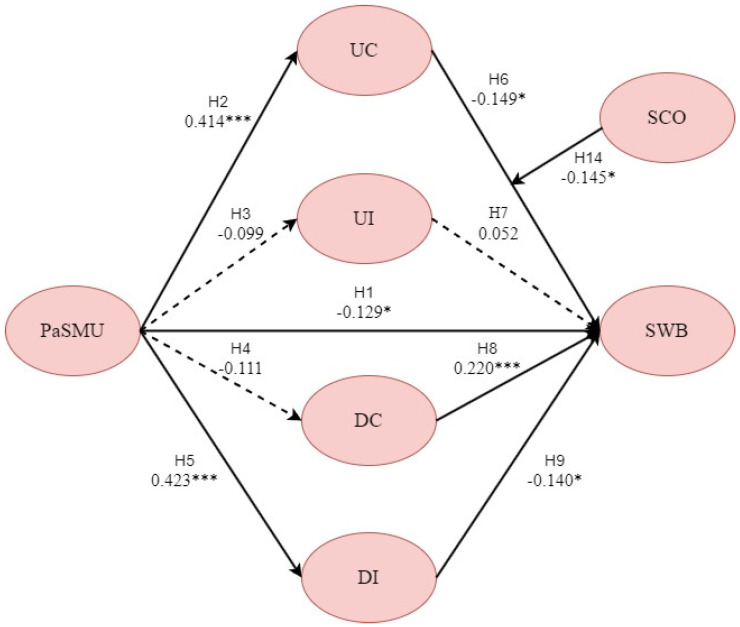
Standardized path diagram. Note: Solid lines indicate significant relationships, while dashed lines represent non-significant relationships; * *p* < 0.05, *** *p* < 0.001. UC = upward contrast; UI = upward identification; DC = downward contrast; DI = downward identification.

The validation of the hypotheses in this study is based on the path coefficients calculated among the variables in the final SEM and is conducted through statistical assessment. The criteria for accepting the hypotheses are that the CR value exceeds ±1.96 and *p* < 0.05. When these criteria are satisfied, the causal relationship of the path is significant (Table 7). The hypotheses involving mediating effects are validated through specific indirect effects (Table 8). The results of the path analysis are illustrated in the structural equation diagram (Figure 4).

### 4.5. ANN Analysis

**Figure 5:**
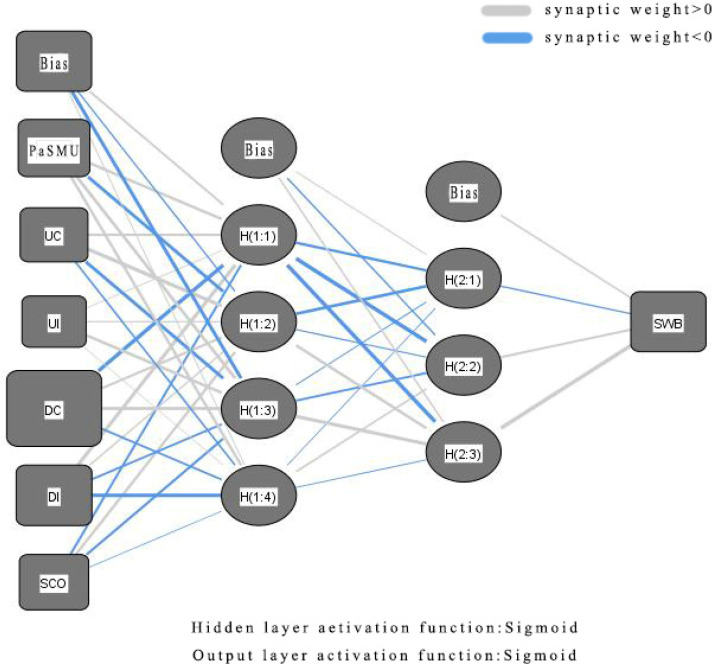
ANN model.

Traditional linear statistical techniques (e.g., MRA and SEM) struggle to adequately simulate the cognitive decision-making of humans [49] due to their reliance on simplified testing via linear model [50]. In contrast, ANN can identify not only linear relationships but also capture intricate nonlinear and non-compensatory relationships without needing to meet assumptions like normality, linearity, or homoscedasticity [47]. Specifically, this study employed a two-layer deep ANN with an MLP structure, automatically determining the number of neurons in the hidden layer (Figure 5).

To assess the predictive capability of the model, we employed ten-fold cross-validation. Specifically, the dataset was randomly divided into ten equal parts, with 90% used as the training set and the remaining 10% for testing. This process was repeated ten times to ensure each part served as the testing set in turn, thus allowing for a comprehensive evaluation of the model’s performance. Through this approach, we calculated the RMSE of the ANN model, yielding RMSE values ranging from 0.227 to 0.375. A low RMSE indicates minimal prediction errors, demonstrating that the model’s predictions on the test data are highly accurate (Table 9).

This study further calculated the normalized importance within the model (Table 10). The results indicate that the ranking of covariates’ normalized importance for the dependent variable is as follows: upward contrast (77.4%), downward contrast (85.5%), and downward identification (86.4%). This ranking aligns closely with the strength relationships of the path coefficients in SEM for upward contrast (−0.149), downward comparison (0.220), and downward identification (−0.140). The nonlinear and non-compensatory characteristics of the neural network model, along with its higher prediction accuracy, account for the subtle differences between the structural equation modeling (SEM) and artificial neural network (ANN) results, thus providing more reliable reference data for understanding the influence of mediating variables on the dependent variable.

## 5. Conclusions and Discussion

Numerous studies have indicated that PaSMU is a key factor contributing to the decline in SWB, primarily because widespread and positive self-presentation on social media can easily trigger upward contrast [32,43]. While previous research has largely focused on upward contrast, there is an urgent need to move beyond the perspective of upward contrast alone to explore other types of social comparison, specifically different patterns based on comparison targets (upward and downward) and comparison methods (identification and contrast). By integrating the theories of social comparison and perceived control, we analyze the relationships among PaSMU, social comparison experiences, SCO, and SWB among college students on the RED platform. The results indicate that PaSMU is significantly related to both upward contrast and downward identification, with these types of comparisons further impacting SWB. Additionally, SCO has a significant effect on social comparison experiences and their outcomes. This study provides a new perspective on the mechanisms by which social media affects mental health and has practical implications.

The findings of this study reveal a significant and positive correlation between PaSMU and both upward contrast and downward identification, which aligns with the results of Yue et al. [51]. Based on prior research that primarily focused on upward contrast, this study further emphasizes that browsing information posted by others not only triggers comparisons with those in better living conditions but also encourages college students to identify with those in worse situations. Passive browsing of diverse social media content leads college students to engage in downward identification, causing them to worry that their circumstances are gradually approaching those unfavorable situations. This may relate to the conditions under which PaSMU occurs; for instance, Cheng et al. [52] found that individuals with social anxiety tend to spend more time and energy on PaSMU. In summary, socially anxious college students are more sensitive to negative information during PaSMU and are prone to emotional resonance. In anxious situations, witnessing the difficulties faced by others can trigger excessive worry and deepen concerns about the deterioration of their own circumstances. Additionally, downward identification may play other roles in emotional regulation; it can assist college students in more actively recognizing and avoiding potential risks by enhancing cognitive alertness, but it may also increase their psychological burden.

This study also explores the underlying mechanisms of the relationship between social media use and SWB. The results reaffirm the mediating role of upward contrast between PaSMU and SWB (as noted by Wang et al. [43]). Notably, this study also finds that downward identification is another significant mediating factor predicting SWB from social media use, with its specific indirect effect strength (β = −0.059) being very similar to that of upward contrast (β = −0.062). Furthermore, normalized importance analysis confirms that downward identification (86.4%) is even more critical in influencing SWB than upward contrast (77.4%). In other words, browsing the activities of others on RED leads college students to identify with those in worse situations, subsequently lowering their SWB. This may arise from emotional contagion triggered by negative content on social media, especially during prolonged exposure to the circumstances of those who are worse off, which is more likely to evoke negative emotions. Research indicates that ongoing contact between users and platform content enhances the effects of emotional contagion [53], while the algorithm of RED promotes similar content, further amplifying emotional resonance and increasing psychological burden. On the other hand, compared to Instagram or Weibo, RED features distinct attributes of “lifestyle sharing” and “shopping recommendations”, leading college students to frequently compare their own “life status” while browsing. In this context, downward identification manifests as a form of “negative prediction”, where users seeing others in difficult situations may inadvertently worry about similar issues happening to themselves, thus experiencing a psychological pressure similar to “preparing for the worst”, which intensifies negative emotions [54]. Moreover, some studies have suggested that depressive symptoms may precede and predict an increase in social media use; however, the reverse causal relationship—where depressive symptoms drive increased social media use—has often been overlooked [27,55]. Therefore, this study acknowledges that lower levels of well-being may lead individuals to engage in passive social media use more frequently, rather than PaSMU being the sole cause of reduced well-being [10]. The possibility of reverse causality cannot be ruled out, necessitating further research for validation.

The mediating role of downward identification suggests another possible explanation, revealing the effects of low self-disclosure in online settings. Compared to other social media platforms, RED incorporates more anonymity features, allowing college students to express their emotions more freely without the concern of impacting their relationships with acquaintances. However, this anonymity also makes it easier for users to overlook the differences in experiences when browsing, leading to the effect of an “imagined community” [7]. Consequently, users often empathize with anonymous content depicting worse situations, which can negatively affect their SWB. This study found that the effects of downward comparison and upward identification on SWB were not significant. The possible reasons are as follows: First, while downward comparison may provide a certain sense of superiority, it is often accompanied by negative emotions such as guilt or pity, which weaken its positive impact on SWB. Second, upward identification is typically a superficial emotional experience based on admiration, lacking deep emotional resonance, making it difficult to exert a lasting and profound influence on well-being. Furthermore, RED’s emphasis on showcasing refined and idealized lifestyles fails to offer concrete pathways or inspiration for personal growth, further diminishing the positive effect of upward identification on SWB.

Numerous studies have confirmed the complex relationship between individuals’ SCO and well-being [40], yet a consistent conclusion regarding the specific mechanisms remains unclear. Shi et al. [56] found that emotions triggered by different comparison orientations can positively influence mental health and well-being. However, this study revealed that SCO amplifies the negative impact of upward contrast on SWB. Specifically, the interaction term between upward contrast and SCO is significantly negatively correlated with SWB (*β* = −0.145, *p* < 0.01). College students with high SCO are more susceptible to the influence of idealized portrayals by others during upward contrasts, resulting in a heightened sense of “self-difference” [57] which increases psychological stress and decreases well-being. On the other hand, college students with high SCO are more inclined to seek short-term social feedback (such as likes and comments) through upward contrast. While this may provide a sense of satisfaction, it also deepens their reliance on external validation, undermining the independence of their self-worth and ultimately leading to negative emotions that impact their well-being [58].

## 6. Limitations and Directions of Future Research

This study primarily relies on self-reported data, which may cause measurement bias. For example, self-reported data often struggle to accurately reflect actual media usage [59]. Future research could combine online browsing records and experience sampling to capture PaSMU and its effects on the emotions and attitudes of users more accurately. Secondly, due to the cross-sectional design of this study, it is challenging to draw causal conclusions. Future research could employ experimental or longitudinal designs to clarify the temporal sequence and causal relationships between variables. Additionally, this study did not sufficiently consider the characteristics of different social media platforms. Users’ responses to platforms may vary based on platform type, content characteristics, and content orientation [60]. Therefore, future research should examine how the differences among various social media platforms influence user behavior in detail. Finally, the direct correlation between PaSMU and SWB suggests that there may be other mediating factors or mechanisms beyond social comparison experiences, which require further empirical investigation. For instance, future studies could explore the dynamics of different social comparison strategies and how these mechanisms affect users’ experiences and mental health.

A significant limitation of this study is the omission of control variables such as students’ academic year, anxiety levels, self-disclosure tendencies, socioeconomic status, and self-esteem, all of which could potentially influence the relationships explored. For instance, students at different academic stages may experience varying levels of stress and social comparison tendencies, moderating the effects of social media use on SWB. Similarly, anxiety levels and self-esteem could shape how individuals interact with social media, influencing their comparison strategies and well-being. Socioeconomic status might also play a role, as individuals from different socioeconomic status backgrounds may engage with social media differently or interpret social comparison outcomes in distinct ways. Self-disclosure tendencies may act as mediators or moderators, further complicating the relationship between social media use and SWB. Future research should incorporate these factors to gain a more nuanced understanding of the underlying mechanisms.

## Figures and Tables

**Table 1:** Reliability and validity test for all variables and items.

Variable	Item	Factor Loading	Cronbach’s α	CR	AVE
PaSMU	I am not very active on RED.	0.826	0.888	0.896	0.632
	I rarely comment on posts or statuses on RED.	0.864			
	I often browse content on RED but do not post updates.	0.771			
	I seldom interact with others on RED.	0.762			
	I am relatively passive on RED.	0.750			
UC	When I see people on RED who are better off than I am, I sometimes feel like I am not living my life well.	0.859	0.826	0.783	0.553
	When I see people on RED who are better off than I am, I feel frustrated because I cannot live like they do.	0.597			
	When I see people on RED who are better off than I am, I feel regret about my current life situation.	0.735			
UI	When I see people on RED who are better off than I am, I feel that my life will also improve like theirs.	0.839	0.813	0.782	0.547
	When I see people on RED who are better off than I am, I believe that things around me will also get better like they have for them.	0.647			
	When I see people on RED who are better off than I am, I feel that my situation will also improve.	0.727			
DC	When I see people on RED who are having a harder time than I am, I feel that I am doing quite well.	0.701	0.811	0.796	0.573
	When I see people on RED who are having a harder time than I am, I feel reassured that I am better off than they are.	0.643			
	When I see people on RED who are having a harder time than I am, I feel that I will gain confidence in my life.	0.883			
DI	When I see people on RED who are having a harder time than I am, I worry that my life will also become as difficult as theirs.	0.785	0.826	0.802	0.578
	When I see people on RED who are having a harder time than I am, I fear that my life will develop in the same way.	0.647			
	When I see people on RED who are having a harder time than I am, I worry that I will also lead an unfortunate life.	0.825			
SWB	I feel very satisfied with my recent life.	0.778	0.898	0.886	0.565
	I believe that most aspects of my life meet my expectations and ideals.	0.748			
	If I could start my university life over, I would hardly make any changes.	0.764			
	Using RED makes me feel energized and creative.	0.701			
	Using RED brings me joy and fulfillment.	0.786			
	Using RED gives me a sense of hope.	0.725			
SCO	I often compare the situations of my close ones (boyfriend, girlfriend, family, etc.) with those of others.	0.768	0.880	0.904	0.705
	I always pay close attention to the differences between how I do things and how others do things.	0.903			
	I often compare my social performance (e.g., social skills, popularity) with that of others.	0.882			
	I frequently compare my achievements in life with those of others.	0.781			

Note: CR = composite reliability; AVE = average variance extracted.

**Table 2:** Results of the discriminant validity test.

	AVE	PaSMU	UC	UI	DC	DI	SWB	SCO
PaSMU	0.632	0.795						
UC	0.553	0.204	0.744					
UI	0.547	0.090	−0.322	0.740				
DC	0.573	0.126	−0.177	0.291	0.757			
DI	0.578	0.115	0.292	−0.370	−0.299	0.760		
SWB	0.565	−0.296	−0.309	0.305	0.307	−0.410	0.752	
SCO	0.705	−0.273	−0.497	0.383	0.223	−0.213	0.531	0.840

Note: The numbers on the diagonal represent the square roots of the AVE for each factor.

**Table 3:** SEM fitting of measured values.

Indicator	CMIN/DF	GFI	CFI	AGFI	RMSEA
Suggested value	1–3	>0.9	>0.9	>0.8	<0.08
Measured value	2.174	0.842	0.908	0.814	0.058

**Table 4:** Specific indirect effects.

Relationships	Point Estimation	Product of Coef.		Bootstrapping			
				BC 95% CI		Percentile 95% CI	
		SE	Z	Lower	Upper	Lower	Upper
Indirect Effects							
PaSMUtoUCtoSWB	−0.062	0.029	−2.138	−0.127	−0.013	−0.122	−0.010
PaSMUtoUItoSWB	−0.005	0.009	−0.556	−0.033	0.004	−0.025	0.010
PaSMUtoDCtoSWB	−0.025	0.019	−1.316	−0.068	0.008	−0.065	0.010
PaSMUtoDItoSWB	−0.059	0.027	−2.185	−0.120	−0.012	−0.113	−0.006
TOTAL	−0.151	0.045	−3.356	−0.252	−0.068	−0.244	−0.063
Contrasts							
UC-UI	−0.057	0.030	−1.900	−0.121	−0.002	−0.121	−0.002
UC-DC	−0.037	0.033	−1.121	−0.100	0.031	−0.102	0.029
UC-DI	−0.003	0.041	−0.073	−0.085	0.077	−0.087	0.076
UC-DI	0.035	0.031	1.129	−0.031	0.092	−0.030	0.092

Note: BC = bias corrected; Z = point estimation/SE; 5000 bootstrap samples; UC = upward contrast; UI = upward identification; DC = downward contrast; DI = downward identification.

**Table 5:** Path analysis of upward contrast and the interaction term of SCO.

Path Analysis							
Path			Unstandardized Coefficient	Standardized Coefficient	S.E.	C.R.	*p*
UC	→	SWB	−0.149	−0.170	0.063	−2.376	0.017
SCO	→	SWB	0.217	0.242	0.061	3.559	***
SCO*UC	→	SWB	−0.145	−0.210	0.050	−2.892	0.004

Note: UC = upward contrast; *** *p* < 0.001; SCO*UC represents the interaction term of Social Comparison Orientation (SCO) and Upward Contrast (UC).

**Table 6:** The test of simple moderation effect after SCO grouping.

Group	Point Estimation	BC 95% CI		*p*
		Lower	Upper	
High SCO	−0.380	−0.599	−0.170	0.000
Mean SCO	−0.170	−0.309	−0.030	0.018
Low SCO	0.040	−0.155	0.242	0.701

**Table 7:** Results of hypotheses for path analysis.

Hypothesis	Relationship Description	Estimate	SE	CR	*p*	Validity
H1	PaSMU→SWB	−0.129	0.063	−2.059	0.040	Y
H2	PaSMU→UC	0.414	0.070	5.914	***	Y
H3	PaSMU→UI	−0.099	0.069	−1.426	0.154	N
H4	PaSMU→DC	−0.111	0.069	−1.604	0.109	N
H5	PaSMU→DI	0.423	0.072	5.891	***	Y
H6	UC→SWB	−0.149	0.063	−2.376	0.017	Y
H7	UI→SWB	0.052	0.059	0.887	0.375	N
H8	DC→SWB	0.220	0.057	3.854	***	Y
H9	DI→SWB	−0.140	0.059	−2.376	0.018	Y
H14	SCO*UC→SWB	−0.145	0.050	−2.892	0.004	Y

Note: *** *p* < 0.001. UC = upward contrast; UI = upward identification; DC = downward contrast; DI = downward identification; SCO*UC represents the interaction term of Social Comparison Orientation (SCO) and Upward Contrast (UC).

**Table 8:** Results of hypotheses for mediating effects.

Hypothesis	Relationship Description	Estimate	S.E.	*p*	Validity
H10	PaSMU→UC→SWB	−0.062	0.029	<0.05	Y
H11	PaSMU→UI→SWB	−0.005	0.009	>0.05	N
H12	PaSMU→DC→SWB	−0.025	0.019	>0.05	N
H13	PaSMU→DI→SWB	−0.059	0.027	<0.05	Y

Note: UC = upward contrast; UI = upward identification; DC = downward contrast; DI = downward identification.

**Table 9:** RMSE of the ANN model.

Input: PaSMU, UC, UI, DC, DI, SCO		
Output: SWB		
Neural Network	Training	Testing
ANN1	0.344	0.375
ANN2	0.307	0.267
ANN3	0.323	0.266
ANN4	0.313	0.375
ANN5	0.292	0.348
ANN6	0.298	0.317
ANN7	0.324	0.304
ANN8	0.319	0.312
ANN9	0.371	0.227
ANN10	0.321	0.324
Mean	0.321	0.312
SD	0.151	0.219

Note: UC = upward contrast; UI = upward identification; DC = downward contrast; DI = downward identification.

**Table 10:** Normalized importance analysis in the ANN model.

Model A (Output: SWB)						
Neural Network	PaSMU	UC	UI	DC	DI	SCO
ANN1	0.146	0.111	0.079	0.355	0.183	0.126
ANN2	0.051	0.126	0.141	0.205	0.180	0.297
ANN3	0.114	0.177	0.038	0.227	0.218	0.226
ANN4	0.068	0.166	0.058	0.171	0.220	0.317
ANN5	0.086	0.184	0.123	0.218	0.147	0.243
ANN6	0.135	0.193	0.093	0.151	0.165	0.263
ANN7	0.153	0.207	0.066	0.180	0.190	0.204
ANN8	0.105	0.111	0.066	0.174	0.240	0.303
ANN9	0.062	0.361	0.040	0.124	0.287	0.126
ANN10	0.091	0.179	0.084	0.204	0.202	0.241
Average relative importance	0.101	0.182	0.079	0.201	0.203	0.235
Normalized relative importance (%)	43.0%	77.4%	33.6%	85.5%	86.4%	100%

Note: UC = upward contrast; UI = upward identification; DC = downward contrast; DI = downward identification.

## Data Availability

The data presented in this study are available on request from the corresponding author due to the interviewees’ request for the conversation content to be kept private.
